# Supplementary material for: Pyruvate Dehydrogenase Kinase 1 inhibition mediated oxidative phosphorylation enhancement in cartilage promotes osteoarthritis progression
Source: BMC Musculoskelet Disord. 2023 Jul 20;24:597. doi: 10.1186/s12891-023-06585-6 (PMC10357736; doi:10.1186/s12891-023-06585-6)
Supplement: Supplementary file 1 — Additional file 1. [file 12891_2023_6585_MOESM1_ESM.zip › MF.Gsea.1653627316242/GOMF_OXIDOREDUCTASE_ACTIVITY_ACTING_ON_PEROXIDE_AS_ACCEPTOR.html]

Details for gene set GOMF\_OXIDOREDUCTASE\_ACTIVITY\_ACTING\_ON\_PEROXIDE\_AS\_ACCEPTOR[GSEA]

|  || Dataset | 55457\_collapsed\_to\_symbols.cls.cls #OA\_versus\_normal.cls.cls #OA\_versus\_normal\_repos |
| Phenotype | cls.cls#OA\_versus\_normal\_repos |
| Upregulated in class | OA |
| GeneSet | GOMF\_OXIDOREDUCTASE\_ACTIVITY\_ACTING\_ON\_PEROXIDE\_AS\_ACCEPTOR |
| Enrichment Score (ES) | 0.3519276 |
| Normalized Enrichment Score (NES) | 1.350881 |
| Nominal p-value | 0.06295399 |
| FDR q-value | 0.3586895 |
| FWER p-Value | 1.0 |
Table: GSEA Results Summary

  

Fig 1: Enrichment plot: GOMF\_OXIDOREDUCTASE\_ACTIVITY\_ACTING\_ON\_PEROXIDE\_AS\_ACCEPTOR      
 Profile of the Running ES Score & Positions of GeneSet Members on the Rank Ordered List

  

| SYMBOL | TITLE | RANK IN GENE LIST | RANK METRIC SCORE | RUNNING ES | CORE ENRICHMENT || 1 | PRDX4 | peroxiredoxin 4 [Source:HGNC Symbol;Acc:HGNC:17169] | 68 | 0.959 | 0.0523 | Yes |
| 2 | PTGS1 | prostaglandin-endoperoxide synthase 1 [Source:HGNC Symbol;Acc:HGNC:9604] | 163 | 0.845 | 0.0957 | Yes |
| 3 | LTC4S | leukotriene C4 synthase [Source:HGNC Symbol;Acc:HGNC:6719] | 223 | 0.804 | 0.1394 | Yes |
| 4 | GSTK1 | glutathione S-transferase kappa 1 [Source:HGNC Symbol;Acc:HGNC:16906] | 490 | 0.662 | 0.1585 | Yes |
| 5 | MGST3 | microsomal glutathione S-transferase 3 [Source:HGNC Symbol;Acc:HGNC:7064] | 610 | 0.620 | 0.1864 | Yes |
| 6 | GSTZ1 | glutathione S-transferase zeta 1 [Source:HGNC Symbol;Acc:HGNC:4643] | 974 | 0.523 | 0.1896 | Yes |
| 7 | GSTT1 | glutathione S-transferase theta 1 [Source:HGNC Symbol;Acc:HGNC:4641] | 1129 | 0.491 | 0.2071 | Yes |
| 8 | CLIC2 | chloride intracellular channel 2 [Source:HGNC Symbol;Acc:HGNC:2063] | 1214 | 0.473 | 0.2290 | Yes |
| 9 | GSTP1 | glutathione S-transferase pi 1 [Source:HGNC Symbol;Acc:HGNC:4638] | 1260 | 0.465 | 0.2534 | Yes |
| 10 | PARK7 | Parkinsonism associated deglycase [Source:HGNC Symbol;Acc:HGNC:16369] | 1406 | 0.442 | 0.2687 | Yes |
| 11 | SELENOF | selenoprotein F [Source:HGNC Symbol;Acc:HGNC:17705] | 1450 | 0.436 | 0.2915 | Yes |
| 12 | PRDX2 | peroxiredoxin 2 [Source:HGNC Symbol;Acc:HGNC:9353] | 1613 | 0.411 | 0.3036 | Yes |
| 13 | ALOX5AP | arachidonate 5-lipoxygenase activating protein [Source:HGNC Symbol;Acc:HGNC:436] | 1665 | 0.404 | 0.3238 | Yes |
| 14 | CAT | catalase [Source:HGNC Symbol;Acc:HGNC:1516] | 1731 | 0.394 | 0.3424 | Yes |
| 15 | GPX7 | glutathione peroxidase 7 [Source:HGNC Symbol;Acc:HGNC:4559] | 1919 | 0.370 | 0.3501 | Yes |
| 16 | PRDX3 | peroxiredoxin 3 [Source:HGNC Symbol;Acc:HGNC:9354] | 2161 | 0.343 | 0.3519 | Yes |
| 17 | MGST2 | microsomal glutathione S-transferase 2 [Source:HGNC Symbol;Acc:HGNC:7063] | 2583 | 0.292 | 0.3367 | No |
| 18 | PRDX1 | peroxiredoxin 1 [Source:HGNC Symbol;Acc:HGNC:9352] | 3113 | 0.239 | 0.3099 | No |
| 19 | GPX5 | glutathione peroxidase 5 [Source:HGNC Symbol;Acc:HGNC:4557] | 3558 | 0.198 | 0.2873 | No |
| 20 | PTGES | prostaglandin E synthase [Source:HGNC Symbol;Acc:HGNC:9599] | 4399 | 0.121 | 0.2292 | No |
| 21 | SESN1 | sestrin 1 [Source:HGNC Symbol;Acc:HGNC:21595] | 5204 | 0.056 | 0.1700 | No |
| 22 | GPX2 | glutathione peroxidase 2 [Source:HGNC Symbol;Acc:HGNC:4554] | 5808 | 0.008 | 0.1236 | No |
| 23 | TPO | thyroid peroxidase [Source:HGNC Symbol;Acc:HGNC:12015] | 6467 | -0.040 | 0.0748 | No |
| 24 | PRDX6 | peroxiredoxin 6 [Source:HGNC Symbol;Acc:HGNC:16753] | 6783 | -0.061 | 0.0540 | No |
| 25 | GPX4 | glutathione peroxidase 4 [Source:HGNC Symbol;Acc:HGNC:4556] | 7115 | -0.089 | 0.0336 | No |
| 26 | PXDN | peroxidasin [Source:HGNC Symbol;Acc:HGNC:14966] | 7642 | -0.130 | 0.0005 | No |
| 27 | IPCEF1 | interaction protein for cytohesin exchange factors 1 [Source:HGNC Symbol;Acc:HGNC:21204] | 7800 | -0.141 | -0.0032 | No |
| 28 | LPO | lactoperoxidase [Source:HGNC Symbol;Acc:HGNC:6678] | 8298 | -0.178 | -0.0311 | No |
| 29 | HBB | hemoglobin subunit beta [Source:HGNC Symbol;Acc:HGNC:4827] | 8775 | -0.215 | -0.0553 | No |
| 30 | HBA1 | hemoglobin subunit alpha 1 [Source:HGNC Symbol;Acc:HGNC:4823] | 8789 | -0.216 | -0.0433 | No |
| 31 | MPO | myeloperoxidase [Source:HGNC Symbol;Acc:HGNC:7218] | 9792 | -0.301 | -0.1032 | No |
| 32 | HBQ1 | hemoglobin subunit theta 1 [Source:HGNC Symbol;Acc:HGNC:4833] | 10473 | -0.370 | -0.1339 | No |
| 33 | EPX | eosinophil peroxidase [Source:HGNC Symbol;Acc:HGNC:3423] | 10492 | -0.372 | -0.1129 | No |
| 34 | HBZ | hemoglobin subunit zeta [Source:HGNC Symbol;Acc:HGNC:4835] | 10615 | -0.385 | -0.0993 | No |
| 35 | DUOX2 | dual oxidase 2 [Source:HGNC Symbol;Acc:HGNC:13273] | 10885 | -0.415 | -0.0953 | No |
| 36 | HBE1 | hemoglobin subunit epsilon 1 [Source:HGNC Symbol;Acc:HGNC:4830] | 11153 | -0.451 | -0.0890 | No |
| 37 | HBG1 | hemoglobin subunit gamma 1 [Source:HGNC Symbol;Acc:HGNC:4831] | 11391 | -0.485 | -0.0783 | No |
| 38 | GPX3 | glutathione peroxidase 3 [Source:HGNC Symbol;Acc:HGNC:4555] | 11407 | -0.488 | -0.0502 | No |
| 39 | GSTA1 | glutathione S-transferase alpha 1 [Source:HGNC Symbol;Acc:HGNC:4626] | 11608 | -0.517 | -0.0347 | No |
| 40 | DUOX1 | dual oxidase 1 [Source:HGNC Symbol;Acc:HGNC:3062] | 12336 | -0.687 | -0.0500 | No |
| 41 | HBD | hemoglobin subunit delta [Source:HGNC Symbol;Acc:HGNC:4829] | 12482 | -0.741 | -0.0168 | No |
| 42 | PTGS2 | prostaglandin-endoperoxide synthase 2 [Source:HGNC Symbol;Acc:HGNC:9605] | 12641 | -0.820 | 0.0201 | No |
Table: GSEA details [plain text format]

  

Fig 2: GOMF\_OXIDOREDUCTASE\_ACTIVITY\_ACTING\_ON\_PEROXIDE\_AS\_ACCEPTOR      
 Blue-Pink O' Gram in the Space of the Analyzed GeneSet

  

Fig 3: GOMF\_OXIDOREDUCTASE\_ACTIVITY\_ACTING\_ON\_PEROXIDE\_AS\_ACCEPTOR: Random ES distribution      
 Gene set null distribution of ES for **GOMF\_OXIDOREDUCTASE\_ACTIVITY\_ACTING\_ON\_PEROXIDE\_AS\_ACCEPTOR**
